# Observation management of pulmonary embolism and agreement with claims-based and clinical risk stratification criteria in United States patients: a retrospective analysis

**DOI:** 10.1186/s12890-017-0379-5

**Published:** 2017-02-13

**Authors:** Elaine Nguyen, Craig I. Coleman, W. Frank Peacock, Philip S. Wells, Erin R. Weeda, Veronica Ashton, Concetta Crivera, Peter Wildgoose, Jeff R. Schein, Thomas J. Bunz, Gregory J. Fermann

**Affiliations:** 1 University of Connecticut School of Pharmacy, 69 North Eagleville Road, Unit 3092, Storrs, CT 06269 USA; 2 Department of Emergency Medicine, Baylor College of Medicine, 1504 Taub Loop, Houston, TX USA; 3 Department of Medicine, University of Ottawa, Ottawa Hospital Research Institute, 501 Smyth Road, Box 206, Ottawa, ON Canada; 4 Janssen Scientific Affairs, LLC, 1000 Route 202, Raritan, NJ USA; 5 New England Health Analytics, LLC, Granby, CT USA; 6 Department of Emergency Medicine, University of Cincinnati, 231 Albert Sabin Way, Cincinnati, OH USA

**Keywords:** Pulmonary embolism, Observation stays, Resource utilization, Mortality

## Abstract

**Background:**

Guidelines suggest observation stays are appropriate for pulmonary embolism (PE) patients at low-risk for early mortality. We sought to assess agreement between United States (US) observation management of PE and claims-based and clinical risk stratification criteria.

**Methods:**

Using US Premier data from 11/2012 to 3/2015, we identified adult observation stay patients with a primary diagnosis of PE, ≥1 PE diagnostic test claim and evidence of PE treatment. The proportion of patients at high-risk was assessed using the In-hospital Mortality for PulmonAry embolism using Claims daTa (IMPACT) equation and high-risk characteristics (age > 80 years, heart failure, chronic lung disease, renal or liver disease, high-risk for bleeding, cancer or need for thrombolysis/embolectomy).

**Results:**

We identified 1633 PE patients managed through an observation stay. Despite their observation status, IMPACT classified 46.4% as high-risk for early mortality and 33.3% had ≥1 high-risk characteristic. Co-morbid heart failure, renal or liver disease, high-risk for major bleeding, cancer and hemodynamic instability were low (each <4.5%), but 7.8% were >80 years-of-age and 19.4% had chronic lung disease.

**Conclusion:**

Many PE patients selected for management in observation stay units appeared to have clinical characteristics suggestive of higher-risk for mortality based upon published claims-based and clinical risk stratification criteria.

## Background

Current guidelines suggest low-risk pulmonary embolism (PE) patients are candidates for treatment at home or with an abbreviated hospital stay versus ~5+ days of in-hospital management [1]. Prior studies suggest up to 50% of PE patients may be treated in such a fashion [2].

In the United States (US), observation stays are intended to manage patients for short periods to determine appropriateness for inpatient admission, with the determination of admission/discharge occurring within 2-midnights. Observation stays may serve as an alternative to inpatient management for low-risk PE patients. Reimbursement policy changes have led to an increasing use of observation stays [3–6]. We assessed agreement between observation management of PE and claims-based and clinical risk stratification criteria using administrative claims data.

## Methods

We performed a retrospective analysis using Premier claims data from 11/2012 to 3/2015. Premier captures ~20% of all discharges from US acute care hospitals. This study was performed in accordance with the Declaration of Helsinki. All data included in the Premier Perspective Comparative Hospital Database are de-identified and are in compliance with the Health Insurance Portability and Accountability Act (HIPAA) of 1996 to preserve participant anonymity and confidentiality. For this reason, this study was exempt from institutional review board oversight and did not require an approval by an ethics committee. The data used in this study was under license from Premier Inc. and provided through the study sponsor, Janssen Scientific Affairs, LLC.

To be included, adult patients had to have undergone an observation stay (self-reported by hospitals) with an International Classification of Diseases, 9^th^-revision (ICD-9) diagnosis code for PE (415.1x) in the primary position, have a claim for ≥1 diagnostic test for PE on day 0–2 (computed tomography, ventilation-perfusion scan, pulmonary angiography) and received pharmacologic (anticoagulation, thrombolysis) and/or non-pharmacologic (pulmonary embolectomy, filter placement) PE treatment [1].

Patient risk for early post-PE mortality was assessed using the validated In-hospital Mortality for PulmonAry embolism using Claims daTa (IMPACT) equation [1/(1 + exp(−x)); x = −5.833 + (0.026 × age) + (0.402 × myocardial infarction) + (0.368 × chronic lung disease) + (0.464 × stroke) + (0.638 × prior major bleeding) + (0.298 × atrial fibrillation) + (1.061 × cognitive impairment) + (0.554 × heart failure) + (0.364 × renal failure) + (0.484 × liver disease) + (0.523 × coagulopathy) + (1.068 × cancer)], with an estimated in-hospital mortality risk >1.5% deemed higher-risk [7–9]. We also determined the proportion of patients with characteristics placing them at higher-risk for early mortality or other complications included in the simplified PE severity index (sPESI) or Hestia criteria (age > 80 years, heart failure, chronic lung disease, renal impairment, liver disease, high-risk for major bleeding, cancer or a need for early thrombolysis/embolectomy [proxy for hemodynamic instability]) when assessable in the claims database [10, 11].

IMPACT mortality risk and patient characteristics were summarized using descriptive statistics. Percentages and means ± standard deviations or medians (25%, 75% range) were used to summarize categorical and continuous data. Statistical analysis was performed in IBM SPSS v22 (IBM Corp., Armonk, NY, USA).

## Results

In total, 47,607 hospital encounters for PE were identified and 3.4% were coded as observation stays. Using IMPACT, ~46% of observation stay patients were at higher-risk for early post-PE mortality (Table 1). Over one-third of PE patients managed via an observation stay had ≥1 criteria placing them at high-risk for early mortality. The proportion of patients with co-morbid heart failure, renal or liver disease, high-risk for major bleeding, cancer and requiring thrombolysis/pulmonary embolectomy were low (<4.5% for each), but 7.8% patients were >80 years-of-age and 19.4% had chronic lung disease. One patient died during hospitalization (out-of-hospital mortality is not available in Premier) and 28% stayed >2-midnights. High-risk patients by IMPACT or those with ≥1 high-risk criteria had longer length-of-stay (*p* ≤ 0.005 for both using a generalized linear model with a gamma distribution and log-link).

**Table 1:** Characteristics of pulmonary embolism patients managed through observation stays

Characteristic	IMPACT low-risk patients, n (%) *N* = 876	IMPACT higher-risk patients, n (%) *N* = 757	No high-risk criteria patients, n (%) *N* = 1,090	≥1 High-risk criteria patients, n (%) *N* = 543
Age (mean ± SD)	43.7 ± 12.3	70.1 ± 10.7	52.3 ± 15.8	63.4 ± 18.5
Male gender	385 (43.9)	340 (44.9)	503 (46.1)	222 (40.9)
High-risk criteria included in sPESI or Hestia criteria				
Age >80 years	0 (0)	127 (16.8)	0 (0)	127 (23.4)
Heart failure	4 (0.5)	68 (9.0)	0 (0)	72 (13.3)
Chronic lung disease	91 (10.4)	226 (29.9)	0 (0)	317 (58.4)
Renal impairment	3 (0.3)	67 (8.9)	0 (0)	70 (12.9)
Severe liver disease	6 (0.7)	23 (3.0)	0 (0)	29 (5.3)
High risk for major bleeding^a^	1 (0.1)	9 (1.2)	0 (0)	10 (1.8)
Cancer	0 (0)	46 (6.1)	0 (0)	46 (8.5)
Required thrombolysis or embolectomy (days 0–2)	2 (0.2)	2 (0.3)	0 (0)	4 (0.7)
Estimated IMPACT^b^ mortality risk, % (mean ± SD)	1.0 ± 0.3	3.2 ± 2.5	1.3 ± 0.7	3.4 ± 2.9
Myocardial infarction	1 (0.1)	0 (0)	0 (0)	1 (0.2)
Stroke	0 (0)	2 (0.3)	0 (0)	2 (0.4)
Atrial Fibrillation	3 (0.3)	85 (11.2)	31 (2.8)	57 (10.5)
Cognitive impairment	0 (0)	57 (7.5)	14 (1.3)	43 (7.9)
Coagulopathy	1 (0.1)	35 (4.6)	18 (1.7)	18 (3.3)
Length of stay, days (mean ± SD) (median [25%, 75% range])	2.1 ± 0.8 2.0 (2.0, 3.0)	2.4 ± 1.6 2.0 (2.0, 3.0)	2.2 ± 1.3 2.0 (2.0, 3.0)	2.3 ± 1.0 2.0 (2.0, 3.0)

*IMPACT* In-hospital Mortality for PulmonAry embolism using Claims daTa, *SD* standard deviation, *sPESI* simplified pulmonary embolism severity index

^a^Active or recent history of major bleed

^b^The multivariable IMPACT equation is: 1/(1 + exp(−x); where x = −5.833 + (0.026*age) + (0.402*myocardial infarction) + (0.368*chronic lung disease) + (0.464*stroke) + (0.638*prior major bleeding) + (0.298*atrial fibrillation) + (1.061*cognitive impairment) + (0.554*heart failure) + (0.364*renal failure) + (0.484*liver disease) + (0.523*coagulopathy) + (1.068*cancer)

## Discussion

Our study suggest anywhere from one-third to about half of PE patients managed via an observation stay in the US would be classified as higher-risk for early post-PE mortality according to claims-based or clinical risk stratification criteria [7–11]. Advanced age and chronic lung disease were the most frequent “high-risk” characteristics identified in observation stay-managed patients.

The substantial proportion of observation stay patients in our study classified at higher-risk by IMPACT and select components of the sPESI and the Hestia criteria highlights the lack of agreement between US observation management of PE and risk stratification tools. Whether this observed disagreement suggests inappropriate use of observation stays is unknown. It does suggest; however, that risk stratification tools may be used infrequently by practitioners in routine practice [12–14]. Studies have reported common clinician barriers to using risk stratification tools including difficulties in implementation in routine practice [12, 13], lack of training in their use, regulatory constraints and the nature of the physician-patient relationship [14]. Also of concern, risk stratification tools tend to oversimplify risk assessment (accurately identifying those at very low-risk, but classify a large majority who inevitably do not experience a complication as higher-risk) [12]. This overall lack of prognostic accuracy likely explains some inconsistencies between tool recommendations and clinical gestalt.

Whether clinical gestalt alone is optimal for identifying PE patients suitable for abbreviated stays is unclear. In a meta-analysis by Piran et al. [15], the pooled risk of recurrent venous thromboembolism in outpatient-treated PE patients was shown not to differ between clinical gestalt and risk stratification methods (1.9% vs. 1.4%). Of note, nearly all studies examined in this meta-analysis were conducted in non-US centers decreasing its generalizability to US practice. Future studies to determine the prognostic accuracy of clinical gestalt in comparison to accepted risk stratification tools are needed.

Our study has limitations worth noting. First, Premier does not provide access to clinical data including vital signs (e.g., blood pressure, heart or respiratory rate, oxygen saturation), out-of-hospital mortality or clot burden. Thus, we could not apply commonly used clinical criteria, tools such as PESI or assess all criteria included in the sPESI and Hestia tools. Inclusion of these data could have only increased the percentage of patients classified as higher-risk, and as a result, we likely underestimated the percentage of patients at high-risk of early post-PE mortality. However, nearly half of observation stay patients in our analysis were identified as high-risk according to the IMPACT equation, which was specifically designed for identification of low-risk PE patients using claims data and shown to have similar prognostic accuracy for both in-hospital and 30-day mortality as PESI, sPESI and Hestia [7–9]. Second, we did not have 30-day mortality data available and therefore cannot comment on whether these observation stay and higher-risk patients had subsequent adverse outcomes after discharge. Finally, since the overall proportion of patients being managed through observations stays in the US is limited, it is possible that other unidentified factors (e.g., societal or economic) are influencing the generalizability of these findings.

## Conclusion

Our analysis suggests patient selection for abbreviated stays is not consistent with claims-based risk stratification tools and accepted clinical criteria denoting higher-risk for early mortality.

## Abbreviations

ICD-9: International Classification of Diseases 9^th^-revision
IMPACT: In-hospital Mortality for PulmonAry embolism using Claims daTa
PE: Pulmonary embolism
SPESI: Simplified pulmonary embolism severity index
US: United States
